# Emergent Surgical Airway Skills: Time to Re-evaluate the Competencies

**DOI:** 10.7759/cureus.23260

**Published:** 2022-03-17

**Authors:** Mohamed Fayed, Katherine Nowak, Santhalakshmi Angappan, Nimesh Patel, Fawaz Abdulkarim, Donald H Penning, Anoop K Chhina

**Affiliations:** 1 Anesthesiology, Pain Management and Perioperative Medicine, Henry Ford Health System, Detroit, USA; 2 Research, Henry Ford Health System, Detroit, USA

**Keywords:** adverse event, crna clinical training, procedure training, skills and simulation training, difficult airway management, adverse respiratory events, quality improvement and patient safety, scalpel cricothyroidotomy, surgical airway, can't intubate can't ventilate

## Abstract

Introduction

One of the most challenging scenarios an anesthesia provider can face is treating a can't intubate can't ventilate (CICV) patient. The incidence of CICV is estimated to be around one in 10,000 cases. According to the American Society of Anesthesiology Closed Claims Study, adverse respiratory events are the most common type of injury, with difficult intubation and ventilation contributing to the majority of these cases. The objective of this non-interventional quality improvement project was to evaluate the prior training, exposure, and self-reported confidence in handling the CICV scenario among anesthesia providers at Henry Ford Hospital in Detroit, MI.

Methods

An online questionnaire was distributed via email to all residents, certified registered nurse anesthetists (CRNAs), and attending anesthesiologists in March 2021. The email contained a link to an online questionnaire via Microsoft Forms (Microsoft Corporation, Redmond, WA). Univariate group comparisons were carried out between the respondents’ role (attending, CRNA, or resident), as well as between the number of years that the respondents were in practice (< 5 years, 5-10 years, > 10 years).

Results

Out of the total 170 anesthesia providers, 119 participated in the study where 54 (45%) were attendings, 44 (37%) were residents, and 21 (18%) were CRNAs. The majority (75%) did not know the surgical airway kit location, and 87% had not performed the surgical airway procedure before. The vast majority (96.7%) recommended simulation training compared to online training or lecture series, and just over 50% recommended annual training frequency. When looking at the differences in responses based on years of experience as an anesthesia provider, the majority of those with > 10 years in practice knew how to perform the surgical airway technique while respondents with < 5 years did not know how to perform the technique, and 50% of those with five to 10 years experience knew how to perform the surgical airway procedure for a CICV scenario.

Conclusion

Although there were many significant differences observed between the various provider roles and years in practice, surprisingly, the responses revealed both a lack of experience and confidence in performing the surgical airway procedure in all provider roles. These findings highlight a need for better emergency airway teaching and training. These findings will be used to guide the design and implementation of improved surgical airway training for residents, CRNAs, and attending anesthesiologists with the goal of better preparedness for handling a CICV scenario.

## Introduction

One of the most challenging scenarios an anesthesia provider can face is treating a can't intubate can't ventilate (CICV) patient. CICV is defined as the inability to intubate a patient's trachea (even after only a single failed attempt) and an inability to maintain arterial oxygen saturation with either a bag and mask or a supraglottic device. Despite marked improvements in airway management in the last decade, the incidence of CICV hasn’t changed over the years; this can be attributed to the lack of specificity and low predictive value of current techniques for predicting when a patient will have a difficult airway [1]. In a survey from 2011 to 2015, a study investigating emergency medical services reported that of the 57,209 patients requiring advanced airway management, 0.5% (286 patients) underwent cricothyroidotomy [2]. In another 10-year survey, a national emergency airway registry indicated that among 17,583 adult intubations, 0.14% (25 patients) received a primary surgical airway and 0.31% (55 patients) received a rescue surgical airway [3]. During the COVID-19 pandemic, respiratory-associated causes have been the leading cause of death among COVID-19 patients [4]. In a study that looked at 4,476 emergency tracheal intubations in suspected or confirmed COVID-19 patients, it was found that the surgical airway technique was used in 0.22% [5].

When the CICV situation arises during emergency airway management, the clinician must take immediate action to avoid a critical drop in oxygen saturation (i.e., < 80%), as this can result in hypoxic brain injury. The American Society of Anesthesiology Closed Claims Study found that adverse respiratory events constituted the single largest injury (522 of 1541 cases, 34%). Three mechanisms of injury accounted for three-fourths of the adverse respiratory events: inadequate ventilation (196 patients; 38%), esophageal intubation (94 patients; 18%), and difficult tracheal intubation (87 patients; 17%); death or brain damage occurred in 85% of cases [6]. The initial surgical airway protocol for a CICV patient includes standard open surgical cricothyrotomy, cricothyrotomy, and cannula-over-needle cricothyrotomy with or without jet ventilation with 100% oxygen [7]. Scalpel cricothyroidotomy is the fastest and most reliable method of securing the airway in the emergency setting [8-9]. These surgical airway procedures are also the recommended final life-saving treatments in the emergent CICV scenario by both the American Society of Anesthesiologists (ASA) and the Difficult Airway Society [10-11].

The objective of this non-interventional quality improvement project was to evaluate the prior training, exposure, and self-confidence in handling the CICV scenario in anesthesia providers at Henry Ford Hospital in Detroit, MI. This was accomplished by distributing an emailed questionnaire to all anesthesia providers that asked about their prior training and exposure to handling a CICV scenario, self-reported confidence in managing this kind of situation, and preference of format for a CICV training initiative. These responses will guide the design and implementation of appropriate educational interventions to improve preparedness for managing CICV patients.

## Materials and methods

This study was approved by the Henry Ford Health System Institutional Review Board on October 28, 2020 (IRB #14788). This study was internally funded by the Henry Ford Health System Department of Anesthesiology, Pain Management & Perioperative Medicine. This project was designed and carried out in accordance with the SQUIRE (Standards for Quality Improvement Reporting Excellence) guidelines. 

To determine the extent of prior training, self-reported competency, and previous exposure to the CICV scenario in anesthesia providers at Henry Ford Hospital, an email describing the study was sent to all residents, certified registered nurse anesthetists (CRNAs), and attending anesthesiologists in March 2021. The email contained a link to an online questionnaire via Microsoft Forms (Microsoft Corporation, Redmond, WA). A consent script preceded the questionnaire, and completion of the questionnaire served as consent to participate in the survey. All survey responses were anonymized, and completion of the survey was voluntary. The questionnaire asked about the training and experience of the provider, their self-reported confidence and ability in performing CICV, and what their preference of CICV training would be.

The sample size was limited to the number of anesthesiology residents, CRNAs, and anesthesiology attendings at Henry Ford Hospital, a total of 176 potential subjects, so a formal a priori sample size calculation was not performed.

All categorical data were reported as counts and column percentages (N (%)). Univariate group comparisons were carried out between the respondents’ role (attending, CRNA, resident), as well as between the number of years that the respondents were in practice (< 5 years, 5-10 years, > 10 years) using the chi-square or Fisher’s exact test for categorical variables. Statistical significance was set at p < 0.05. All analyses were performed using SAS 9.4 (SAS Institute Inc., Cary, NC).

## Results

**Figure 1:**
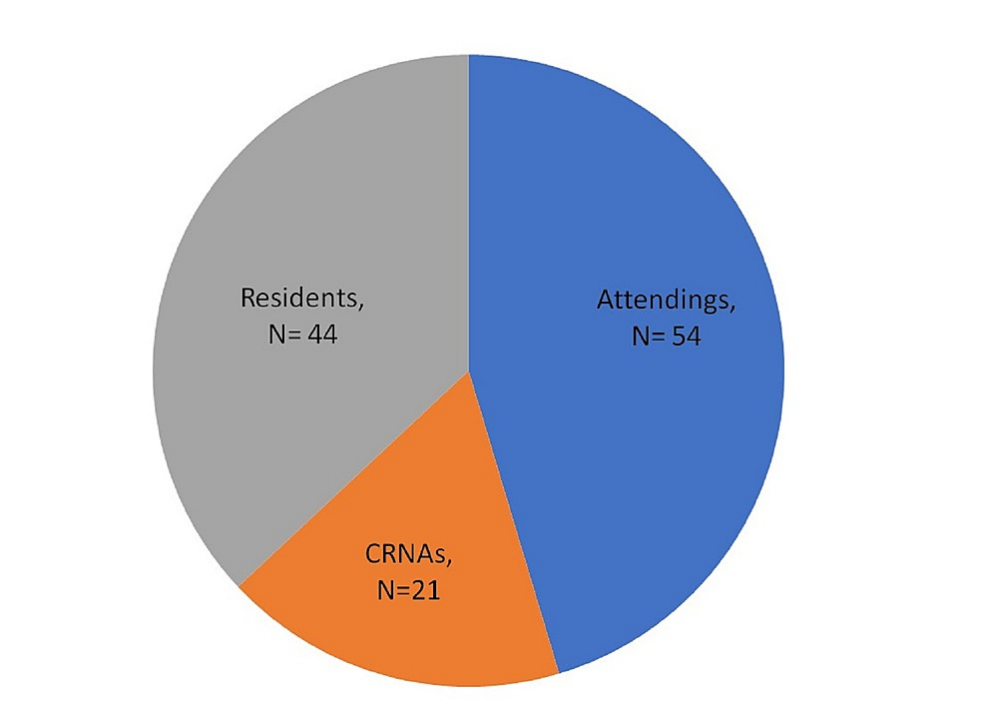
Distribution of participating anesthesia providers CRNA: certified registered nurse anesthetist

Out of the 176 total anesthesia providers at Henry Ford Hospital, 119 (67.6%) participated in the study by responding to the questionnaire. Of the respondents, 54 (45%) were attendings, 44 (37%) residents, and 21 (18%) CRNAs (Figure 1). Of the participants, 53% were in practice for less than five years and 52% did not know how to perform the emergent surgical airway procedure. The majority (75%) did not know where the surgical airway kit is located, and 87% have not performed the surgical airway procedure before. The vast majority recommended annual simulation training (96.7%) as part of an annual training experience compared to online training or a lecture series provided less frequently or as needed (Table 1).

**Table 1:** Descriptive statistics for all survey responses N: number, %: percent, CICV: can't intubate can't ventilate

Survey Question	Level	Frequency (%) N = 119
What is your role in anesthesia?	Attending	54 (45.4)
	CRNA	21 (17.6)
	Resident	44 (37.0)
How many years have you been in practice?	5-10	21 (17.6)
	<5	63 (52.9)
	>10	35 (29.4)
Do you know how to perform the surgical airway procedure for handling a CICV scenario?	No	61 (51.7)
	Yes	57 (48.3)
	Missing	1
Do you know where the CICV kit is located?	No	90 (75.6)
	Yes	29 (24.4)
Have you been trained in the surgical airway technique for a CICV scenario?	No	59 (50.4)
	Yes	58 (49.6)
	Missing	2
Have you performed the surgical airway technique during a CICV scenario?	No	80 (87.0)
	Yes	12 (13.0)
	Missing	27
How much do you agree with the following statement: "I am confident in performing the surgical airway technique during a CICV scenario"	Agree	6 (5.0)
	Disagree	71 (59.7)
	Neither agree nor disagree	15 (12.6)
	Missing	27 (22.7)
What would be your optimal teaching method for learning and/or refreshing your skills in the surgical airway technique for handling a CICV scenario?	Lecture	1 (1.1)
	Online course	2 (2.2)
	Simulation training	89 (96.7)
	Missing	27
What would be your desired frequency of refreshing your skills in this method?	Annually	48 (52.2)
	As needed	16 (17.4)
	Every 2 years	28 (30.4)
	Missing	27

Univariate comparison between different roles

**Figure 2:**
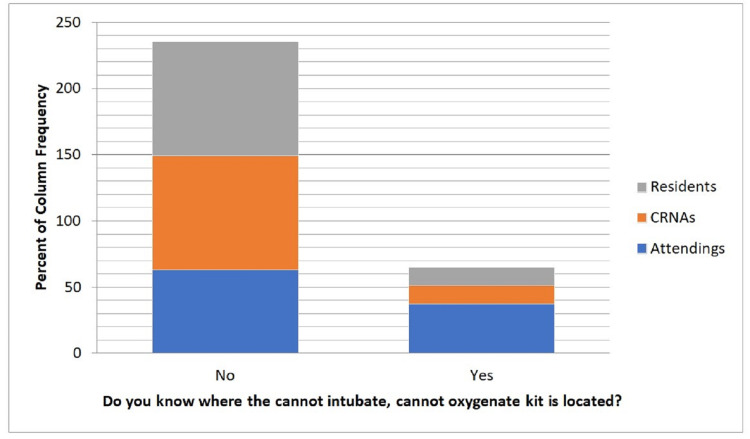
Awareness of the location of the can't intubate can't ventilate kit CRNA: certified registered nurse anesthetist

**Figure 3:**
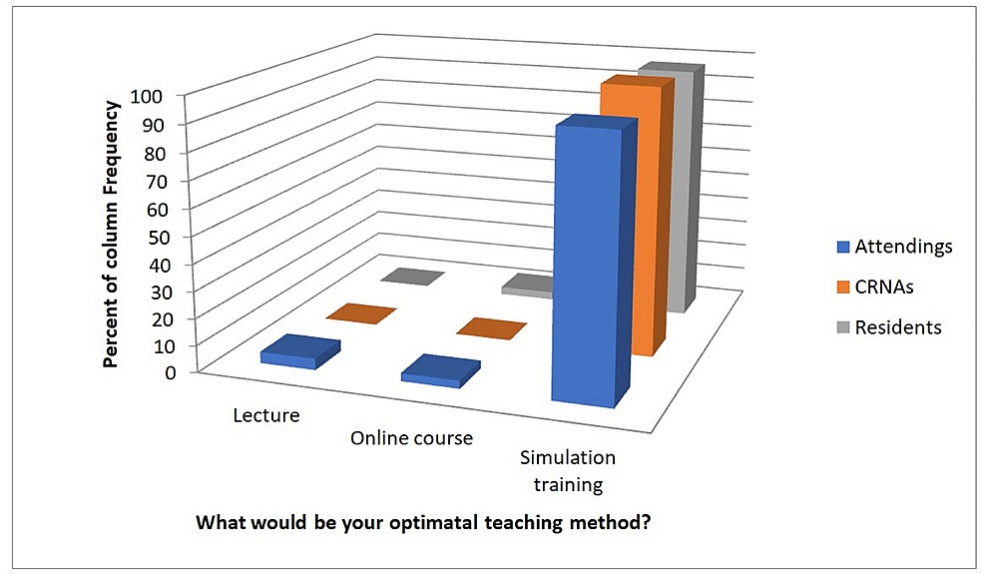
Desired teaching method CRNA: certified registered nurse anesthetist

When a univariate comparison was made to evaluate the differences in responses between the three provider roles (CRNA, resident, or attending), significant differences between the groups were observed for the number of years in practice, knowledge of how to perform the surgical airway procedure for CICV, knowledge of where the CICV kit is, prior training in the CICV scenario, previous performance of the surgical airway technique during a CICV scenario, and confidence in performing the surgical airway technique in a CICV scenario (Table 2). Overall, the attendings who responded were in practice longer than residents and CRNAs. Of the attendings, the majority knew how to perform the surgical airway technique while the majority of residents and CRNAs did not know how to perform the technique. Across all roles, the majority did not know where to locate the CICV kit (Figure 2). More attendings have been trained in the surgical airway technique than CRNAs and residents. Across all groups, most providers had not performed the surgical airway technique during a CICV scenario, and most do not feel confident in performing the surgical airway technique in a CICV scenario. There was not a significant difference in the preferred optimal teaching method or preferred frequency of CICV training between the different provider roles. The majority of all respondents preferred annual simulation training (Figure 3), with the exception of attendings, where 50% of attendings had indicated that they would prefer training every two years.

**Table 2:** Survey responses compared across respondent’s role in anesthesiology %: percent, N: number, CICV: can't intubate can't ventilate

			Role		
Covariate	Frequency	Level	Attending N=54	CRNA N=21	Resident N=44	P-value
How many years have you been in practice?	N (Col %)	5-10	13 (24.07)	4 (19.05)	4 (9.09)	< .001>
	N (Col %)	<5	15 (27.78)	9 (42.86)	39 (88.64)	
	N (Col %)	>10	26 (48.15)	8 (38.1)	1 (2.27)	
Do you know how to perform the surgical airway procedure for handling a CICV?	N (Col %)	No	11 (20.75)	12 (57.14)	38 (86.36)	< .001>
	N (Col %)	Yes	42 (79.25)	9 (42.86)	6 (13.64)	
Do you know where the CICV kit is located?	N (Col %)	No	34 (62.96)	18 (85.71)	38 (86.36)	0.016
	N (Col %)	Yes	20 (37.04)	3 (14.29)	6 (13.64)	
Have you been trained in the surgical airway technique for a CICV scenario?	N (Col %)	No	13 (25)	12 (57.14)	34 (77.27)	< .001>
	N (Col %)	Yes	39 (75)	9 (42.86)	10 (22.73)	
Have you performed the surgical airway technique during a CICV scenario?	N (Col %)	No	28 (75.68)	19 (90.48)	33 (97.06)	0.023
	N (Col %)	Yes	9 (24.32)	2 (9.52)	1 (2.94)	
How much do you agree with the following statement: "I am confident in performing the surgical airway technique during a CICV scenario"	N (Col %)	Agree	6 (11.11)	0 (0)	0 (0)	< .001>
	N (Col %)	Disagree	19 (35.19)	19 (90.48)	33 (75)	
	N (Col %)	Neither agree nor disagree	12 (22.22)	2 (9.52)	1 (2.27)	
What would be your optimal teaching method for learning and/or refreshing your skills in the surgical airway technique for handling a CICV scenario?	N (Col %)	Lecture	1 (2.7)	0 (0)	0 (0)	1.000
	N (Col %)	Online course	1 (2.7)	0 (0)	1 (2.94)	
	N (Col %)	Simulation training	35 (94.59)	21 (100)	33 (97.06)	
What would be your desired frequency of refreshing your skills in this method?	N (Col %)	Annually (at beginning of training year)	15 (40.54)	15 (71.43)	18 (52.94)	0.237
	N (Col %)	As needed	7 (18.92)	2 (9.52)	7 (20.59)	
	N (Col %)	Every 2 years (with renewal of ACLS certification)	15 (40.54)	4 (19.05)	9 (26.47)	

Univariate comparison using years of experience

When a univariate comparison was made to evaluate the differences in responses between the three categories of years in practice (<5, 5-10, or >10), significant differences between the groups were observed regarding the provider’s role in anesthesiology (resident, CRNA, or attending), knowledge of how to perform the surgical airway procedure for CICV, knowledge of where the CICV kit is, prior training for the CICV scenario, the prior performance of the surgical airway technique during a CVCI scenario, and confidence in their ability to perform the surgical airway technique in a CICV scenario (Table 3). When looking at the differences in responses based on years of experience as an anesthesia provider, the majority of those with > 10 years in practice knew how to perform the surgical airway technique while respondents with < 5 years do not know how to perform the technique, and those with five to 10 years are 50/50 on knowing how to perform the surgical airway procedure for a CICV scenario (Table 3). Across all years, the majority still do not know where to locate the CICV kit. The majority of those with at least five years in practice have been trained in the surgical airway technique while the majority of respondents with < 5 years in practice have not been trained in the technique. Across all groups, most have not performed the surgical airway technique during a CICV scenario, and most do not feel confident in performing the surgical airway technique in a CICV scenario (Table 3). All three groups preferred annual simulation training as an optimal teaching method.

**Table 3:** Survey responses compared across respondent’s number of years in practice %: percent, N: number, CICV: can't intubate can't ventilate

		Frequency (%)			
		Years in Practice			
Question	Response	<5 years, N=63	5-10 years, N=21	>10 years, N=35	P-value
What is your role in anesthesia?	Attending	15 (23.81)	13 (61.9)	26 (74.29)	< .001>
	CRNA	9 (14.29)	4 (19.05)	8 (22.86)	
	Resident	39 (61.9)	4 (19.05)	1 (2.86)	
Do you know how to perform the surgical airway procedure for handling a CICV scenario?	No	44 (69.84)	10 (50)	7 (20)	< .001>
	Yes	19 (30.16)	10 (50)	28 (80)	
Do you know where the CICV kit is located?	No	51 (80.95)	18 (85.71)	21 (60)	0.040
	Yes	12 (19.05)	3 (14.29)	14 (40)	
Have you been trained in the surgical airway technique for a CICV scenario?	No	41 (66.13)	7 (35)	11 (31.43)	0.001
	Yes	21 (33.87)	13 (65)	24 (68.57)	
Have you performed the surgical airway technique during a CICV scenario?	No	49 (96.08)	14 (87.5)	17 (68)	0.003
	Yes	2 (3.92)	2 (12.5)	8 (32)	
How much do you agree with the following statement: "I am confident in performing the surgical airway technique during a CICV scenario"	Agree	1 (1.59)	0 (0)	5 (14.29)	0.013
	Disagree	45 (71.43)	13 (61.9)	13 (37.14)	
	Neither agree nor disagree	5 (7.94)	3 (14.29)	7 (20)	
What would be your optimal teaching method for learning and/or refreshing your skills in the surgical airway technique for handling a CICV scenario?	Lecture	0 (0)	0 (0)	1 (4)	0.472
	Online course	1 (1.96)	0 (0)	1 (4)	
	Simulation training	50 (98.04)	16 (100)	23 (92)	
What would be your desired frequency of refreshing your skills in this method?	Annually	27 (52.94)	8 (50)	13 (52)	0.366
	As needed	12 (23.53)	2 (12.5)	2 (8)	
	Every 2 years =	12 (23.53)	6 (37.5)	10 (40)	

## Discussion

This analysis revealed significant differences in anesthesia providers’ self-reported experience with CICV and confidence in their own ability to perform the surgical airway technique based on whether they are a resident, CRNA, or attending, as well as the number of years in practice. However, a surprisingly high number of respondents in all categories expressed a lack of training, experience, and self-confidence in performing this technique, indicating a need for improved education.

Few previous studies have evaluated airway competencies or prior experience and training in anesthesia providers. One investigation found that 90% of residents showed no familiarity with the advanced surgical airway technique [12]. Another study looked at graduating otolaryngology and anesthesiology residents and found more anesthesiology residents have not performed an emergency surgical airway than otolaryngology residents (92% vs 18%). Despite the fact that such a high percentage of residents had never performed an emergency surgical airway, their self-rating of competency was high, with 82% responding with an 8 or higher (10 indicating that they feel “totally competent”) [13].

Among all of the provider types and years of experience evaluated in this study, the optimal training method that was selected was simulation training performed annually. A previous study investigated if participating in bedside elective tracheostomies improves the self-reported competence of anesthesiology residents in performing an emergent invasive airway. The residents reported that this was an essential aspect of their anesthesiology training, but it did not improve their competence in performing an invasive airway [14]. Another study that evaluated web-based or online training indicated a significant improvement (29%) in knowledge following completion of the training, with a 90% recruitment rate and 65% retention rate [15]. An evaluation of the use of problem-based learning among 35 anesthesiology residents did not show significant improvements in knowledge and competency following problem-based learning in emergent airway procedures [16]. When another group of anesthesiology residents practiced emergent surgical airway procedures on preserved cadavers, the number of residents who reported that they would use emergent airway procedures increased from 0% to 78% (P < 0.001) and those who reported that they could correctly perform emergent airway procedure increased from 17% to 94% (P < 0.001) [17].

There were several limitations in this analysis. Some of the inherent drawbacks of using a questionnaire are that this method of data collection allows for subjectivity in responses, and results are susceptible to response bias, as only those who participated are represented in the results. Additionally, the assessments were fully self-reported and may not be reflective of how a provider would handle a real-life CICV scenario. Confidence in performing the emergent airway technique may not necessarily translate into being able to correctly perform the procedure when needed. The self-reporting of this information also relied on an assumption of honesty from respondents. Although the submissions were anonymized, it is possible that respondents were not truthful when answering the questions and inflated their responses regarding experience, training, knowledge, and confidence. It must also be noted that there was a low response rate from the CRNAs in the department. These providers represent a large portion of the anesthesia workforce, so it is critical to have a proper assessment of their experience, training, and confidence in handling a CICV scenario.

## Conclusions

Our project provides insight into how provider role and years in practice are associated with an anesthesiology provider’s prior experience with and confidence in performing the surgical airway procedure in a CICV scenario. Although there were many significant differences observed between the various provider roles and their years in practice, the responses indicate a surprisingly high lack of experience and confidence in all provider roles. This highlights a need for more emergency airway teaching and training. While the debate continues regarding the relative merits and risks of various methods for performing the procedure, it remains clear that skill acquisition and maintenance are vital. We suggest that clinicians who may become responsible for emergent airway management review the anatomy and practice with the equipment needed for cricothyrotomy.
